# Association of Transfer RNA Fragments in White Blood Cells With Antibody Response to Bovine Leukemia Virus in Holstein Cattle

**DOI:** 10.3389/fgene.2018.00236

**Published:** 2018-07-04

**Authors:** Tasia M. Taxis, Marcus E. Kehrli, Rui D’Orey-Branco, Eduardo Casas

**Affiliations:** ^1^National Animal Disease Center, Agricultural Research Service, United States Department of Agriculture, Ames, IA, United States; ^2^Department of Animal Science, Michigan State University, East Lansing, MI, United States; ^3^Department of Animal Science, Texas A&M University, Overton, TX, United States

**Keywords:** BLV, bovine, cattle, leukemia virus, small non-coding RNA, tRNA halves, tRFs

## Abstract

Bovine leukemia virus (BLV) affects cattle health and productivity worldwide, causing abnormal immune function and immunosuppression. Transfer RNA fragments (tRFs) are known to be involved in inhibition of gene expression and have been associated with stress and immune response, tumor growth, and viral infection. The objective of this study was to identify tRFs associated with antibody response to BLV in Holstein cattle. Sera from 14 animals were collected to establish IgG reactivity to BLV by ELISA. Seven animals were seropositive (positive group) and seven were seronegative (negative group) for BLV exposure. Leukocytes from each animal were collected and tRFs were extracted for sequencing. tRF5^GlnCTG^, tRF5^GlnTTG^, and tRF5^HisGTG^, were significantly different between seropositive and seronegative groups (*P* < 0.0067). In all cases the positive group had a lower number of normalized sequences for tRFs when compared to the negative group. Result suggests that tRF5s could potentially be used as biomarkers to establish exposure of cattle to BLV.

## Introduction

Bovine leukemia virus (BLV) is the causative agent of enzootic bovine leucosis that leads to lymphosarcoma in a subset of infected cows and condemnation at slaughter (Rhodes et al., 2003; Frie and Coussens, 2015; Bauermann et al., 2017). The disease has been described in three stages: asymptomatic, persistent lymphocytosis (PL), and leukemia or lymphosarcoma (Bartlett et al., 2013). It has been estimated that BLV incidence in dairy herds is approximately 83% (Frie and Coussens, 2015), while a recent cross-sectional epidemiological survey estimated the incidence to be 39% among 1996 US dairy and beef cattle culled for slaughter (Bauermann et al., 2017). Infection by BLV has been identified as a silent threat to the immune status and health of cattle (Frie and Coussens, 2015). Currently there is no commercial vaccine available for the disease and the most efficient control measure is eliminating positive animals from the herd (Bauermann et al., 2017). Infection with BLV is important to control because is associated with higher cull rates and a shorter herd life, which are factors that contribute to the cost of this disease (Thurmond et al., 1985). The mean annual cost of subclinical BLV infection with a 50% prevalence for every 100 dairy cows is approximately $6,406 (Rhodes et al., 2003).

Bovine leukemia virus infects B-lymphocytes in cattle that typically seroconvert within 3 weeks to 3 months following experimental inoculation (Miller and Van Der Maaten, 1975; Evermann et al., 1986). This leads to 30% or more of the seropositive cattle developing PL (Ferrer et al., 1978). In contrast to PL, development of lymphosarcoma is less frequent (<5% of cattle with PL) and appears to be under independent genetic control. Cows with PL and seropositive cows with normal lymphocyte counts have elevated B cell percentages (Lewin and Bernoco, 1986; Lewin et al., 1988a). The PL results from a polyclonal B-cell expansion. The B-cell transcriptome has been investigated to understand characteristic cytokine messenger RNA (mRNA) expression (Teutsch and Lewin, 1996; Amills et al., 2004) and T-cell changes occurring in cattle infected with BLV (Amills et al., 2002). A correlation between stages of BLV infection with dysfunctional lymphocyte recognition of BLV proteins has been reported (Orlik and Splitter, 1996), thus indicating a loss of T-cell competence as a result of disease progression. In addition, mRNA of cytokines such as interleukin-2 (IL-2), IL-12, and γ-interferon increase in peripheral blood mononuclear cells (PBMCs) of animals during early stages of BLV infection and decrease in PBMCs from animals with later stages of BLV infection (Pyeon et al., 1996; Yakobson et al., 1998). In contrast, IL-10 mRNA expression increased with disease progression (Pyeon et al., 1996). These changes in cytokine mRNA expression and loss of immune cell competence likely contribute to the increase in secondary disease susceptibility and higher rate of culling seen with BLV infected cattle (Thurmond et al., 1985; Trainin et al., 1996; Blagitz et al., 2017).

Understanding differences in the leukocyte transcriptome between BLV infected and non-infected cows and factors affecting those differences may advance our understanding of the pathogenesis of enzootic bovine leucosis. Part of the host transcriptome includes transfer RNA fragments (tRFs) that are short (<40 nucleotides) RNA fragments derived from transfer RNA (tRNA) transcripts. tRFs were originally considered a degradation product of protein translation, but it has been established they have a role in various biological functions through control of gene expression (Garcia-Silva et al., 2012; Sobala and Hutvagner, 2013; Venkatesh et al., 2016). The specific function of tRFs is unknown, but a growing body of evidence indicates that they have similar functions as microRNAs in regulating gene silencing and cell proliferation (Dhahbi, 2015; Venkatesh et al., 2016). tRFs are generated by the cleavage of tRNA molecules in the cell, and are classified based on the site of cleavage. Two types of tRFs, designated as tRF5 and tRF3, are processed from the 5′ or the 3′ end of the mature tRNA, respectively. Those produced from the 3′ end of the immature tRNA are denoted as tRF1 (Lee et al., 2009; Haussecker et al., 2010; Kumar et al., 2014). These three types of tRFs are the most abundant small non-coding RNAs in serum, and the second most abundant in other tissues, after microRNAs (Lee et al., 2009; Dhahbi et al., 2013; Casas et al., 2015; Dhahbi, 2015). In an initial cattle study, tRF5s are more abundant than tRF3s and tRF1s (Casas et al., 2015).

Transfer RNA fragments are involved in the inhibition of gene expression under several conditions, and they have been associated with stress response, immune response, tumor growth, and viral infections (Thompson and Parker, 2009; Ivanov et al., 2011; Garcia-Silva et al., 2012; Wang et al., 2013; Dhahbi, 2015; Goodarzi et al., 2015; Duechler et al., 2016; Green et al., 2016). Recently, certain tRFs have been shown to be induced by specific cleavage of tRNA following infection by respiratory syncytial virus and that tRF5^GluCTC^ was stimulatory to respiratory syncytial virus replication (Wang et al., 2013). In addition, tRF5^GlyCCC^ and tRF5^LysCTT^ were found capable of regulating RSV replication through gene *trans*-silencing by their impact on RSV-induced cytokines and chemokines (Zhou et al., 2017). Given that circulating tRFs may play a role in mediating the infection-induced host response, our objective was to identify tRF5s associated with leukocytes in BLV-infected cattle that had seroconverted to the virus.

## Materials and Methods

### Animals

Samples from 14 Holstein females were collected at the National Animal Disease Center, in Ames, IA, United States. At sampling, 3 animals were heifers and 11 were cows with at least one calving and at mid-lactance. Blood samples were collected via jugular venipuncture in PAXgene tubes (PreAnalytiX GmbH, Hombrechtikon, Zurich, Switzerland) to obtain white blood cells (WBCs). Tubes were incubated at room temperature for 2 h and placed in refrigeration (4°C) before being centrifuged (3000 *g* for 10 min) prior to RNA extraction. Housing, care, and sample collection from animals was done according to the management protocol approved by the Institutional Animal Care and Use Committee of the National Animal Disease Center, in Ames, IA, United States.

### ELISA

A second blood sample was also collected from each animal via jugular venipuncture in serum separator vacutainer tubes (SST,^TM^ BD, Franklin Lakes, NJ, United States) to obtain sera. After collection, tubes were incubated (37°C for 30 min) and centrifuged (1250 *g* for 30 min). Isolated sera were stored at -80°C until processed. Sera were used to establish IgG reactivity to BLV with a direct ELISA, using the IDEXX Leukosis Serum X2 Ab kit (Idexx Laboratories, Westbrook, ME, United States). Positive samples were determined according to manufacturer’s instructions. Briefly, a sample was considered positive if the sample to positive ratio (S/P) was greater than 115%. The S/P was determined by the ratio of the difference between the optical density 450 nm (OD_450_) of the sample minus the OD_450_ of the negative control, divided by the difference between the OD_450_ of the mean positive control minus the OD_450_ of the negative control. From the ELISA, seven animals were identified as seronegative (negative group), and seven were seropositive (positive group).

### RNA Isolation

Total RNA was purified from WBC samples using the MagMAX^TM^ mirVana^TM^ Total RNA Isolation Kit (Life Technologies, Carlsbad, CA, United States) and was eluted in 100 μL of RNase-free water. The concentration and quality of small RNAs in each sample was determined using a 10–40 nucleotide gate on an Agilent 2100 Bioanalyzer Small RNA chip (Agilent Technologies, Santa Clara, CA, United States).

### Library Preparation and Sequencing

Six microliters (6 μL) of small RNA from each extraction was used to prepare individual libraries using the NEBNext Multiplex Small RNA Library Prep Kit (New England BioLabs, Ipswich, MA, United States) and 14 Illumina indexed primers, giving each sample a unique identifier (barcode). Library concentration and purification was performed using the QIAquick PCR purification kit (QIAGEN, Germantown, MD, United States). Each library was run on an Agilent 2100 Bioanalyzer High Sensitivity DNA chip (Agilent Technologies, Santa Clara, CA, United States) to determine quality and quantity of the prepared library between 135 and 170 base pairs. Then, 30 ng of each library was pooled (14 libraries in the pool) and size selected using AMPure XP beads (Beckman Coulter, Indianapolis, IN, United States). Following size selection, library pools were concentrated using the QIAquick PCR purification kit (QIAGEN, Germantown, MD, United States) and eluted in RNase-free water. An Agilent 2100 Bioanalyzer High Sensitivity DNA chip (Agilent Technologies, Santa Clara, CA, United States) was used to determine the concentration of each library pool between 135 and 170 base pairs. The library pool was stored at -20°C until sequencing. The size selected library pool was sequenced as single-end 50 base pair reads using the Illumina HiSeq 3000 System (Illumina, San Diego, CA, United States).

### Data Analysis

FastQC v0.11.2^1^ and fastx_clipper program in a fastx toolkit^2^ was used to determine the quality of the sequences and remove the adapter sequence from each read, respectively. Unique sequences were collapsed using a custom script and sequences 18–40 nucleotides in length were retained for analysis (Baras et al., 2015). These sequences were initially mapped to the *Bos taurus* genome (ENSEMBL UMD3.1.75) using NovoAlign software (Novocraft Technologies), allowing two mismatches. *B. taurus* genome aligned sequences were then aligned to a database containing different annotated genome features in order to determine the aligned sequences’ origin: genomic tRNA sequences were downloaded from the website^3^; mitochondrial tRNA, cDNA, and other non-coding RNA sequences were downloaded from ENSEMBL version 75. The sequences that aligned to tRNA genes or their flanking sequences were further characterized. First, these sequences were aligned to a *B. taurus* tRNA database (see footnote 3) using BLASTN and the results were processed using a custom script. Sequences that aligned perfectly to the 5′ end of a mature tRNA were classified as a tRF5, and sequences that aligned perfectly to the 3′ end of a mature tRNA were classified as a tRF3. Sequences that aligned to the 3′ end of the immature tRNA were classified as tRF1. After tRF5, tRF3, and tRF1 sequences were determined, the number of sequences per WBC sample was obtained using a custom script, and normalization of library size to reads per million (RPM) was obtained for statistical analysis (Casas et al., 2015). Sequences are available on the NCBI SRA under BioProject accession number PRJNA378560.

### Statistical Analysis

**Table 1** shows the number of sequences corresponding to each tRF5 and the anticodon for each amino acid. Those tRF5s with fewer than 500 sequences were excluded from further analysis due to low copy number of sequences (Wilson Van Voorhis and Morgan, 2007; Motameny et al., 2010). All tRF3s and tRF1s were also excluded from further analysis for having a low copy number of sequences, with 764 and 3,770 total sequences for tRF3s and tRF1s, respectively.

**Table 1:** Median size of each 5′ transfer RNA fragment (tRF5) sequences by amino acid and anticodon, number of read counts, significance (*P*-value), normalized means by bovine leukemia virus (BLV) group (positive and negative), and standard errors (SE).

tRNA fragment	Median size (nucleotides)	*N*	*P*-value^a^	BLV		SE
				Positive (RPM)^b^	Negative (RPM)^b^	
tRF5^AlaAGC^	30.5	397	–	–	–	–
tRF5^AlaCGC^	30	2,254	0.22	5.54	7.92	1.29
tRF5^AlaTGC^	30	4	–	–	–	–
tRF5^ArgCCG^	32	10	–	–	–	–
tRF5^ArgTCT^	40	11	–	–	–	–
tRF5^AsnGTT^	50	13	–	–	–	–
tRF5^AspGTC^	33.5	2,118	0.74	6.09	5.65	0.94
tRF5^CysGCA^	32.5	297	–	–	–	–
tRF5^GlnCTG^	33.5	1,927	0.0065	3.71	7.41	0.8
tRF5^GlnTTG^	30	745	0.0003	1.36	2.92	0.22
tRF5^GluCTC^	35.5	37,097	0.24	83	161	45
tRF5^GluTTC^	33	473,291	0.18	1108	1829	359
tRF5^GlyCCC^	32	775,359	0.23	1846	2953	617
tRF5^GlyGCC^	39	94,906	0.54	255	305	55
tRF5^GlyTCC^	32	2,065	0.27	6.47	5.03	0.88
tRF5^HisGTG^	32.5	305,072	0.0062	615	1188	123
tRF5^IleAAT^	32	2	–	–	–	–
tRF5^LeuAAG^	33.5	13	–	–	–	–
tRF5^LeuCAG^	35.5	4	–	–	–	–
tRF5^LeuTAA^	50	4	–	–	–	–
tRF5^LeuTAG^	42	2	–	–	–	–
tRF5^LysCTT^	35	22,788	0.37	58	87	22
tRF5^LysTTT^	33	1,325	0.85	3.55	3.72	0.6
tRF5^MetCAT^	31	17	–	–	–	–
tRF5^ProAGG^	32	3,390	0.0496	7.22	13.47	2.02
tRF5^ProCGG^	34	22	–	–	–	–
tRF5^ProTGG^	47.5	389	–	–	–	–
tRF5^SeCTCA^	36.5	86	–	–	–	–
tRF5^SerCGA^	32	2	–	–	–	–
tRF5^SerGCT^	50	5	–	–	–	–
tRF5^ThrAGT^	50	5	–	–	–	–
tRF5^ThrCGT^	31.5	27	–	–	–	–
tRF5^TrpCCA^	32	3	–	–	–	–
tRF5^TyrGTA^	35	1	–	–	–	–
tRF5^V alAAC^	32	363	–	–	–	–
tRF5^V alCAC^	34.5	28,477	0.24	68	104	21
tRF5^V alTAC^	33.5	4,328	0.03	9.65	14.88	1.55
Total		1,756,819				


*^*a*^Statistically significance at *P* < 0.0067; ^*b*^RPM, Reads per million.*

Data was analyzed as a GLM procedure from SAS (SAS Institute Inc., Cary, NC, United States), with the fixed effect of treatment (positive versus negative group). Significance threshold was adjusted for the number of comparisons using the false discovery rate approach (Benjamini and Hochberg, 1995). A *P* < 0.0067 was considered statistically significant.

Sequence of significant tRF5s were BLASTed against the bovine genome^4^ using the UMD 3.1.1 reference annotation release (Bovine Genome et al., 2009). The objective was to verify their location within the genome and to establish neighboring genes that could potentially be associated with their production. Only tRF5s with 100% homology were included in this study.

For identification purposes, the nomenclature of tRF5s will be by amino acid and anticodon. For example, the tRF5 for alanine will be abbreviated as tRF5^Ala^. When a specific anticodon is being referred to, the anticodon will be included. For tRF5^Ala^, anticodon AGC, the abbreviation tRF5^AlaAGC^ will be used.

## Results

A total of 371,492,458 sequences were obtained from sequencing 14 libraries. Each library resulted with a similar number of sequences (an average of 26,535,176 sequences per library). From these, 3,196,388 sequences mapped to genomic tRNA. Only sequences that matched 100% to tRNA genes and their flanking sequences were included in further analyses. There was a total of 1,761,353 sequences matching these criteria, and 1,756,819 of these sequences were identified as tRF5s (**Table 1**). The tRF5s for each amino acid with the highest number of sequences were tRF5^Glu^ (510,388, total of two anticodons), tRF5^Gly^ (872,330, total of three anticodons), tRF5^His^ (30,572), tRF5^Lys^ (24,113, total of two anticodons), and tRF5^V al^ (33168, total of three anticodons). A total of 15 tRF5s had greater than 500 sequences and were included in further analyses to identify tRF5s associated with BLV status (**Table 1**).

**Table 1** shows the median size of each tRF5. The sizes ranged from 30 to 50 nucleotides, with an overall median of 33.5 nucleotides in length. For the 15 tRF5s included in additional analyses, the median was 33 nucleotides in length, ranging from 30 to 39 nucleotides.

Significance of analyzed tRF5s is shown on **Table 1**. In all cases the positive group had a lower number of normalized sequences when compared to the negative group, and the negative group had approximately double the number of sequences for each tRF5’s when compared to the positive group. The tRNA fragments, tRF5^GlnCTG^, tRF5^GlnTTG^, and tRF5^HisGTG^, were associated with statistical differences between positive and negative groups (**Table 1**).

## Discussion

Human research trials have reported a difference in expression of tRF^Gln^, tRF^His^, and tRF^V al^ associated with acute myeloid leukemia (AML) (Guo et al., 2017). These tRFs are similar to those identified in the present study, where significant differences were observed between positive and negative groups for tRF5^GlnCTG^, tRF5^GlnTTG^, and tRF5^HisGTG^. The present study also observed that cattle in the positive group had an overall lower number of tRF5s compared to the negative group. This has not been reported in other studies evaluating tRFs related to immune response. Guo et al. (2017) showed a greater number of sequences for tRF5s in patients affected by leukemia, when compared to non-affected patients. This discrepancy between human and cattle may be attributed to differences in experimental design. This could be due to different stages of the viral infection, because in the present study, expression of tRFs was measured among cattle exposed to BLV, whereas the human study was completed in patients suffering from fully developed leukemia (Guo et al., 2017). Also, two different tissues were used, with Guo et al. (2017) evaluating bone marrow as opposed to WBC that was evaluated in the present study. Bone marrow has been proposed as the source of tRFs (Nolte-’t Hoen et al., 2012); however, other tissues could also be responsible for tRF production. Irrespective of production site, serum is a rich source of tRFs (Thompson and Parker, 2009; Ivanov et al., 2011; Casas et al., 2015). Regardless of differences in expression, tRF^Gln^ and tRF^His^ were identified in the present study and the study by Guo et al. (2017). The presence of both tRFs in BLV seropositive cattle and leukemia patients warrants further evaluation to potentially establish their role in BLV disease pathogenesis.

Differences in expression of tRFs were also identified *in vitro* (Ruggero et al., 2014). The role of tRFs in human T-cell leukemia virus type-1 (HTLV-1) in CD4+ cells was studied. Ruggero et al. (2014) identified tRF-3019, corresponding to tRF3^ProTGG^ as the only differentially expressed tRF *in vitro*. The tRF3s were excluded from the present study due to low number of sequences (*n* = 592). None of the tRF5^Pro^ (tRF5^ProAGG^, tRF5^ProCGG^, or tRF5^ProTGG^) from the present study were statistically significant, although there was indication that tRF5^ProAGG^ could be associated with BLV. Further studies would be needed to validate this signal. No additional comparison could be established between this study and the report by Ruggero et al. (2014).

Expression of tRF5s have been evaluated in cattle exposed to *Mycoplasma bovis* (Casas et al., 2018). Casas et al. (2018) compared the read counts of tRF5s throughout the growing seasons (summer, fall, and spring of the following year) of beef cattle. Casas et al. (2018), indicated that all but two significant tRF5s have higher read counts in seropositive, when compared to seronegative animals. Differences in counts of tRF5s should not be unexpected. Viruses and bacteria have different colonization mechanisms within an organism, therefore, the genes involved in the defense mechanism of the organism against the pathogen should be different, therefore, the expression of metabolites should also be different (Taxis and Casas, 2017).

Size of sequences obtained in this study had at least 30 nucleotides, which could be considered tRNA halves. Dhahbi (2015), indicate that tRNA fragments are approximately 30 nucleotides in length, being expressed in hematopoietic and lymphoid organs, and circulating in the blood stream as stable molecules. It has also been established that tRNA halves are expressed in immune tissues in the bloodstream, which would be the case of WBCs, and can be modulated by conditions established by pathogens (Dhahbi, 2015). WBCs was the tissue evaluated in the present study instead of serum, so it is likely that tRF5s in the present study correspond to tRNA halves.

The tRNA halves have been associated with diseases (Honda et al., 2015; Nientiedt et al., 2016; Zhao et al., 2018). Angiogenin is a ribonuclease that under stress conditions cleaves tRNA at the anticodon loop, producing tRNA-derived fragments, including tRNA halves (Ivanov et al., 2011; Saikia et al., 2014). It has been established that angiogenin is associated with increased production of tRNA^HisGTG^ in breast cancer cells (Honda et al., 2015). Differences in counts for tRF5^HisGTG^ were observed in the present study. Similarly, differences in expression of tRNA halves were identified in patients affected with clear cell renal cell carcinoma, where tRNA^GlnTTG^ was upregulated in patients affected with the condition (Nientiedt et al., 2016). In the present study tRF5^GlnTTG^ was associated with BLV status. The studies where differences in expression of tRNA halves have been identified, treated or affected subjects have increased amounts, which is different from what was observed in the present study. This difference could potentially be explain because in the present study only animals exposed to BLV, whereas previous studies evaluated tissues or cells affected with the condition. Additional studies would need to be pursued to explain these differences.

Transfer RNAs are dispersed throughout the genome; however, clusters of tRNAs have been identified (Bermudez-Santana et al., 2010). It has been established that not only these clusters play a role in the functional organization of the genome, but they seem to be associated with protein complexes (McFarlane and Whitehall, 2009). A correlation was observed between the location of the tRNAs in the genome with DNA replication (Wyrick et al., 2001). This may lead to consideration of the role of tRF location in specific genomic regions, with transcriptional regulation of neighboring genes.

A tRNA cluster was identified as being the source of tRF5s on chromosome 23, where significant tRF5^GlnCTG^ resided. In the bovine genome, 25% of the tRNAs reside in clusters (Bermudez-Santana et al., 2010), and in the present study, tRF5^GlnCTG^ resided in a cluster. Given that tRNAs are the source of tRFs (Lee et al., 2009), it would not be surprising if the locations of the tRFs have a functional role during nearby replication processes.

The bovine major histocompatibility complex (*BoLA*) has been associated with resistance to PL caused by BLV (Lewin and Bernoco, 1986; Lewin et al., 1988b; Xu et al., 1993). The tRNA cluster, where tRF5^GlnCTG^ resides on chromosome 23, is located within a region of *BoLA* lacking known genes, but within its limits (Brinkmeyer-Langford et al., 2009). Polymorphisms in the *BoLA-DRB3* gene locus have been correlated to resistance for PL due to BLV in cattle (Xu et al., 1993). The *BoLA-DRB3* gene is located between megabases 25.47 and 25.77. This gene is approximately four megabases from the cluster where tRF5^GlnCTG^ resides. Given the location of tRF5^GlnCTG^ within this genomic region, it could be associated with function of neighboring genes as has been proposed (McFarlane and Whitehall, 2009; Bermudez-Santana et al., 2010).

Based on the results of the current study and previously reported studies evaluating tRFs, several tRF5s could be potentially involved in BLV disease progression and pathogenesis leading to PL or lymphosarcoma. The similarities of tRF5 expression between cattle exposed to BLV and human leukemia patients hold potential for tRF5s to be used to identify key regulatory processes of BLV infection in cattle.

## Author Contributions

TT, RD’O-B, and EC conceived and designed the experiment. EC performed the experiment. TT analyzed the data. EC and TT interpreted the results. EC, TT, and MK wrote the manuscript. RD’O-B and MK reviewed and edited the manuscript.

## Conflict of Interest Statement

The authors declare that the research was conducted in the absence of any commercial or financial relationships that could be construed as a potential conflict of interest.

## Footnotes

1 http://www.bioinformatics.babraham.ac.uk/projects/fastqc

2 http://hannonlab.cshl.edu/fastx_toolkit/

3 http://gtrnadb.ucsc.edu/

4 https://www.ncbi.nlm.nih.gov/genome?term=bos%20taurus
